# Effects of *β*-sitosterol derived from *Artemisia capillaris* on the activated human hepatic stellate cells and dimethylnitrosamine-induced mouse liver fibrosis

**DOI:** 10.1186/1472-6882-14-363

**Published:** 2014-09-27

**Authors:** Ki-Suk Kim, Hea Jung Yang, Jae-Youl Lee, Yun-Cheol Na, Soo-Young Kwon, Young-Chul Kim, Jang-Hoon Lee, Hyeung-Jin Jang

**Affiliations:** College of Korean Medicine, Institute of Korean Medicine, Kyung Hee University, 1 Heogi-dong, Dondaemun-gu, Seoul, 130-701 Republic of Korea; Korea Basic Science Institute, 126-16 Anam-Dong, Sungbuk-Gu, Seoul, 136-713 Korea

**Keywords:** *β*-sitosterol, Anti-fibrosis, Collagen type-1 (collagen-1), *α*-smooth muscle actin (*α*-SMA), Dimethylnitrosamine (DMN)

## Abstract

**Background:**

*β*-sitosterol is a cholesterol-like phytosterol, which widely distributed in the plant kingdom. Here, anti-fibrotic effect of the *β*-sitosterol was studied using the activated human hepatic stellate cell (HSC) model and dimethylnitrosamine (DMN)-induced mouse hepatic fibrosis model.

**Method:**

HSCs were activated by transforming growth factor-*β* (TGF-*β*) and the collagen-1 and *α*-smooth muscle actin (*α*-SMA) expressions were measured at the mRNA and protein level. We also studied the effect *β*-sitosterol using DMN-induced mouse hepatic fibrosis model. We then measured the collagen-1 and *α*-SMA expression levels *in vivo* to investigate anti-hepatofibrotic effect of *β*-sitosterol, at both of the mRNA and protein level.

**Results:**

*β*-sitosterol down regulated the mRNA and protein expression levels of collagen-1 and *α*-SMA in activated HSC. Oral administration of the *β*-sitosterol successfully alleviated the DMN-induced mouse liver damage and prevented collagen accumulation. The mRNA and protein expression levels of collagen-1 and *α*-SMA were also down regulated in *β*-sitosterol treated mouse group.

**Conclusions:**

This study shows the effect of *β*-sitosterol on the TGF-*β* -or DMN-induced hepatofibrosis. Hence, we demonstrate the *β*-sitosterol as a potential therapeutic agent for the hepatofibrosis.

## Background

Fibrosis is a wound healing process in which damaged regions are filled with an extracellular matrix (ECM). In liver, chronic injury leading to fibrosis occur in response to a variety of causes, including viral hepatitis, alcohol abuse, drugs, metabolic disease, autoimmune disease, or congenital abnormalities [1–4].

Liver damage provoke cellular changes that stimulate the recruitment of inflammatory cells and activate fibrogenic cells [5]. And these cells secrete different signal molecules that promote ECM accumulation [5].

Fibrogenic cell differentiation and ECM accumulation are usually induced by the transforming growth factor-beta (TGF-*β*) and the platelet-derived growth factor (PDGF) [5]. Induced liver fibrogenesis model have been studied with the TGF-*β*1 gene knock-out mice which showed accumulation of collagen-1 and alpha-smooth muscle actin (*α*-SMA) in their liver tissues [5, 6]. Increased expression level of *α*-SMA is a marker of activated HSC model [5, 7].

Hepatic stellate cells (HSCs), comprise 15% of the total number of resident liver cells, adequate cellular model for investigation of liver fibrosis following their activation into fibrogenic myofibroblast-like cells [8]. Phenotypic responses of activated HSCs include proliferation, contractility, fibrogenesis, matrix degradation, chemotaxis, retinoid loss, cytokine release, and white blood cell chemoattraction [2].

Activated HSCs participate in the synthesis and deposition of the ECM component and the induction of *α*-SMA [9]. Therefore, HSC activation plays a significant role during hepatic fibrosis in response to TGF-*β* through increased synthesis of ECM proteins such as, collagen-1 and *α*-SMA [9].

A water extract of *Artemisia capillaris* (AC) have been transcribed for liver protection in traditional Korean medicine [10]. Here, we found that the *β*-sitosterol is the common active compound of the AC and has a hepatoprotective effect. *β*-sitosterol is a phytosterol, which is widely distributed in the plant kingdom, but only few studies reported its role as a phytomedicine. *β*-sitosterol only have been studied its reducing effect on the blood levels of cholesterol and inhibits cholesterol absorption in the intestine [11].

In this study, anti-fibrotic effect of the *β*-sitosterol is studied in both of the activated HSC model and the dimethylnitrosamine (DMN)-treated mouse model. In experiments, both of the mRNA and protein expression levels of collagen-1 and *α*-SMA are measured, respectively. Gas chromatography/mass spectrometry (GC/MS) results demonstrate the *β*-sitosterol is an active compound of a water extract of AC.

## Methods

### Sample preparation

The aerial part of AC was purchased from Kyung Hee Oriental Herbal Medicine Research Center (Seoul, South Korea). The herb was cut down in a proper size, and extracted with distilled water (DW) for the chromatography analysis as described in references [12, 13]. Briefly, AC was extracted with distilled water (DW) and then filtration, evaporation, and freeze drying were performed in order [14]. The extracts were diluted with DW for appropriate concentrations before use.

The chemical compounds including TGF-*β*1 (PeproTech, Rocky Hill, NJ, USA), DMN (Supelco, Bellefonte, PA, USA), 3-(4, 5-dimethylthiazole-2-yl)-2, 5-diphenyltetrazolium bromide (MTT) (Invitrogen, Carlsbad, CA, USA), and *β*-sitosterol (Santa Cruz Biotechnology, Dallas, TX, USA) were purchased from each manufacturer.

### GC/MS

An Agilent GC/MS system composed of an Agilent 6890 gas chromatograph and an Agilent 5975i mass spectrometer (Agilent Technologies, Palo Alto, CA, USA) was used to identify *β*-sitosterol in AC extract. The extracts dissolved in methanol were injected in split mode (10:1 ratio). The carrier gas was helium (99.999%) with a flow rate of 1 ml/min. The oven was initially held at 50°C for 5 min, increased to 300°C at 15°C/min, and then held at this temperature for 15 min. A DB-5MS cross-linked 5% phenyl methylsilicone fused-silica capillary column (30 m × 0.25 mm i.d., 0.25 μm film thickness) was used to separate the samples. The column was interfaced directly to the electron impact (EI) ion source of the mass spectrometer. The ion source was operated at 70 eV. The injection port, transfer line and ion source temperature were set at 300°C and 230°C, respectively. For the identification of *β*-sitosterol, the retention time and mass spectrum was compared with its standard material and selected ion monitoring (SIM) technique at m/z 414.5 was applied.

### Cell culture

LX-2 cells, human hepatic stellate cell lines, were kindly provided by Dr. Scott Friedman (Icahn Medical Institute, New York, NY, USA). Cells were maintained in Dulbecco’s modified Eagle’s medium (DMEM) containing 4.5 g/ml glucose (Lonza, Allendale, NJ, USA) supplemented with 10% fetal bovine serum (FBS) (Lonza) and Antibiotics & antimycotics (Sigma-Aldrich, St. Louis, MO, USA) in a humidified atmosphere of 5% CO_2_ at 37°C.

### HSC activation and drug treatment

LX-2 cells were plated at 100 mm dishes as 6 × 10^6^ cells per dish. Once they reached 70% confluence, the media was replaced with DMEM supplemented with 0.2% bovine serum albumin (BSA). After 48 h of incubation, cells were incubated with media including each concentration of the *β*-sitosterol for 1 h and incubated further 20 h with media including 5 ng/ml of TGF-*β*1.

### Cell viability assay

Cell viability assay was performed using MTT assay to determine the suitability of each concentration of the *β*-sitosterol on the TGF-*β*1 treated LX-2 cells. Cells were plated at 96-well plates as 4 × 10^4^ cells per well. MTT assay was performed according to the manufacturer’s protocol.

### Real-time quantitative PCR

Total RNA was extracted from the LX-2 cells and the partial rat liver tissues with Ribo Spin kit (GeneAll, Seoul, South Korea). Subsequently, cDNA was hybridized from 1 μg of total RNA with LeGene 1^st^ strand cDNA synthesis system (LeGene bioscience, San Diego, CA, USA). The expression levels of each target mRNA were determined with real-time quantitative PCR using SYBR® PCR master mix (Applied Biosystems, Foster City, CA, USA) as described in manufacturer’s protocol. The 2^-ΔΔCt^ value compared to the normal mouse sample was determined with StepOne software (Applied Biosystems). Human and mouse glyceraldehyde 3-phosphate dehydrogenase (*GAPDH or Gapdh*) genes were used as an endogenous control, respectively. Each PCR primer was designed using Primer express 3.0 software (Applied Biosystems). Oligonucleotide sequences of PCR primers for the target genes are listed in Table 1. The results are from at least two individual experiments performed in triplicate.

**Table 1:** **Real time PCR primer sequence**

Gene		Forward primer (5' → 3')	Reverse primer (5' → 3')
Human	*GAPDH*	CATGGCCTTCCGTGTTCCTA	GCGGCACGTCAGATCCA
	*COL1A1*	GAGACTGTTCTGTTCCTTGTGTAACTG	CCCGGTGACACATCAAGACA
	*ACTA2*	TGCCTGATGGGCAAGTGA	CTGGGCAGCGGAAACG
	*MMP1*	GATCATCGGGACAACTCTCCT	TCCGGGTAGAAGGGATTTGTG
	*MMP2*	TGAGCTATGGACCTTGGGAGAA	CCATCGGCGTTCCCATAC
	*GFAP*	CCGCAGCCCTGAAAGAGA	TTGCTGGACGCCATTGC
Mouse	*Gapdh*	GCCACATCGCTCAGACACC	CCCAATACGACCAAATCCGT
	*Col1a1*	CGATGGCGTGCTATGCAA	ACTCGCCCTCCCGTTTTT
	*Acta2*	CCATGTACCCAGGCATTGCT	GGGAGCGAGGGCTGTGAT

*Abbreviations:* *GAPDH* human glyceraldehyde 3-phosphate dehydrogenase gene, *COL1A1* human collagen type-1 alpha-1 gene, *ACTA2* human alpha-smooth muscle actin gene, *MMP1* human matrix metalloproteinase-1 gene, *MMP2* human matrix metalloproteinase-2 gene, *GFAP* human glial fibrillary acidic protein gene, *Gapdh* mouse glyceraldehyde 3-phosphate dehydrogenase gene, *Col1a1* mouse collagen type-1 alpha-1 gene, *Acta2* mouse alpha-smooth muscle actin gene.

### Western blot

Protein expression levels of *β*-actin, collagen-1 and *α*-SMA from the LX-2 cells and the mouse liver tissues were determined by western blot. Fifty-micrograms of each whole cell lysate samples were subjected to SDS–PAGE. Mouse anti-*β*-actin (1:2000 dilution; Santa Cruz Biotechnology), rabbit anti-collagen-1 (1:1000 dilution; Abcam, Cambridge, UK) and rabbit anti-*α*-SMA (1:200 dilution, Abcam) were used. The reactions were detected with HRP-conjugated secondary antibodies of each host. Blots were developed using ECL detection system (Davinch-Chemi Imaging System; CoreSciences, Seoul, South Korea). *β*-actin expression level was used as equal protein loading control.

### Animals

Five-weeks-old male C57BL/6 mouse weighing between 20 to 25 g were housed individually in cages with a 12 h light-dark cycle and given free access to water and standard chow throughout the study. Twenty-four mice were purchased from Daehan biolink (DBL, Eumseoung-gun, Chungcheongbuk-do, South Korea). All *in vivo* experimental processes were approved by the Kyung Hee University Institutional Animal Care and Use Committee (IACUC).

### Fibrosis model and tissue preparation

Twenty-four mice were separated into 4 groups; the control group (control group), the DMN-treated group received saline (DMN group), and the DMN-treated group received 10- or 40 mg/kg of *β*-sitosterol (*β*-sitosterol group), respectively. Mice were injected intraperitoneally (i.p.) with 10 μg/kg of DMN for three consecutive days per week for up to four weeks [15]. After DMN injections, each mouse group received oral administration of *β*-sitosterol, which appropriately diluted with DW, or saline for two weeks. After two weeks of oral administration, animals were sacrificed and the liver tissues were isolated. Total RNA and protein were extracted directly as soon as the liver samples were excised. Liver tissue samples were fixed in 4% paraformaldehyde (PFA) for two days.

### Immunohistochemistry (IHC)

Mouse liver tissues were embedded to the paraffin to prepare the paraffin section (5 μm). Sections were deparaffinized in xylene and dehydrated with the ethanol series. The sections were incubated with 3% H_2_O_2_ in methanol for 10 min and then, were treated with citrate buffer (pH 6.0) for antigen retrieval. And the sections were incubated with 2.5% normal horse serum in saline for 15 min at room temperature to prevent nonspecific binding of antibodies. Then, the sections were incubated with the following primary antibodies in saline at 4°C for overnight (for *α*-SMA) or at room temperature for 30 min (for collagen-1): rabbit anti-*α*-SMA (1:2000; Abcam), rabbit anti-collagen-1 (1:500; Abcam). After equilibrating to room temperature, sections were incubated with ImmPRESS anti-rabbit Ig (peroxidase) polymer detection kit (Vector Laboratory, Burlingame, CA, USA) for 1 h. Immunostaining was examined by incubation with 3,3’-diaminobenzidine (DAB; Vector Laboratory, Burlingame, CA, USA) and the sections were counterstained with Harris’ hematoxylin (Sigma-Aldrich) and observed using optical microscope (BX61, Olympus, Japan).

### Hematoxylin and eosin (H&E) staining

Tissue samples obtained from the mouse liver were stained with H&E. The histological sections were deparaffinized in xylene, rehydrated through a graded series of ethanol, and washed in running water. The sections were immersed in Harris' hematoxylin for 2 min, washed and stained in an aqueous solution of eosin (Sigma-Aldrich) for 5 min, and dehydrated with the ethanol series. Next, the sections were cleared in xylene (three successive changes, 1 min each) and mounted under a cover slip in DPX Mountant for histology (Sigma-Aldrich).

### Statistical analysis

The results of western blot and the observed images obtained from histological staining were analyzed using ImageJ software (Image J, NIH, USA). The significances of the data were analyzed with Graphpad Prism 5 software (Graphpad software, La Jolla, CA, USA) with one-way ANOVA and Bonferroni’s post-hoc test. The bars show the means ± SEM for triplicate experiments.

## Results

### *β*-sitosterol is an active ingredient of AC water extract

**Figure 1:**
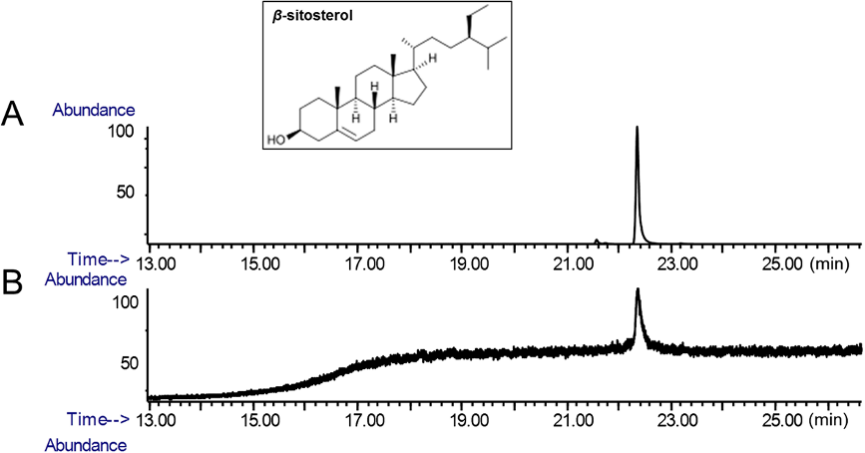
**GC/MS.** Selected ion chromatograms of *β*-sitosterol standard **(A)** and *Artemisia capillaris* water extract **(B)** diluted in methanol.

GC/MS was performed to investigate the active ingredient of a water extract of AC. SIM chromatograms of AC extract targeted with *β*-sitosterol (Figure 1B) was compared to the *β*-sitosterol standard (Figure 1A). The peak observed at 22.2 to 22.6 min of AC extract was identified as a *β*-sitosterol (Figure 1B).

### *β*-sitosterol regulates collagen-1 and α-SMA expression levels in activated HSCs

**Figure 2:**
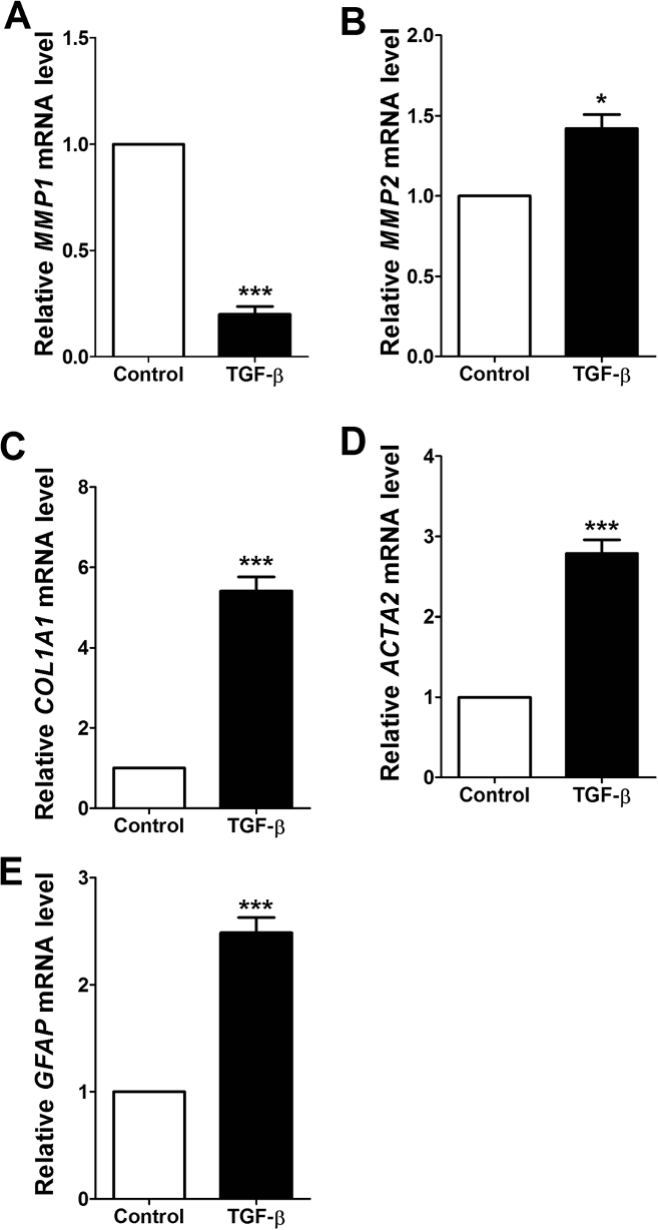
**Effects of TGF-β treatment on the activation of HSCs.** Relatively expressed *MMP1* **(A)**, *MMP2* **(B)**, *COL1A1* **(C)**, *ACTA2* **(D)**, and *GFAP* **(E)** mRNA levels were measured by real-time quantitative PCR. Experiments were carried out at least twice performed in triplicate. Statistical significance determined by one-way ANOVA; values are means ± SEM; *, *p* < 0.05; ***, *p* < 0.001 vs control group.

To investigate the anti-fibrotic effect of the *β*-sitosterol, we induced the activated HSC model with TGF-*β*-treated LX-2 cells. We have confirmed the suitability of the activated HSC model with real-time PCR targeted with several activated HSC markers, those are down regulation of matrix metalloproteinase (MMP)-1 mRNA level (Figure 2A), and up regulations of MMP-2, collagen-1, *α*-SMA, and glial fibrillary acidic protein (GFAP) mRNA levels (Figure 2B-E).

**Figure 3:**
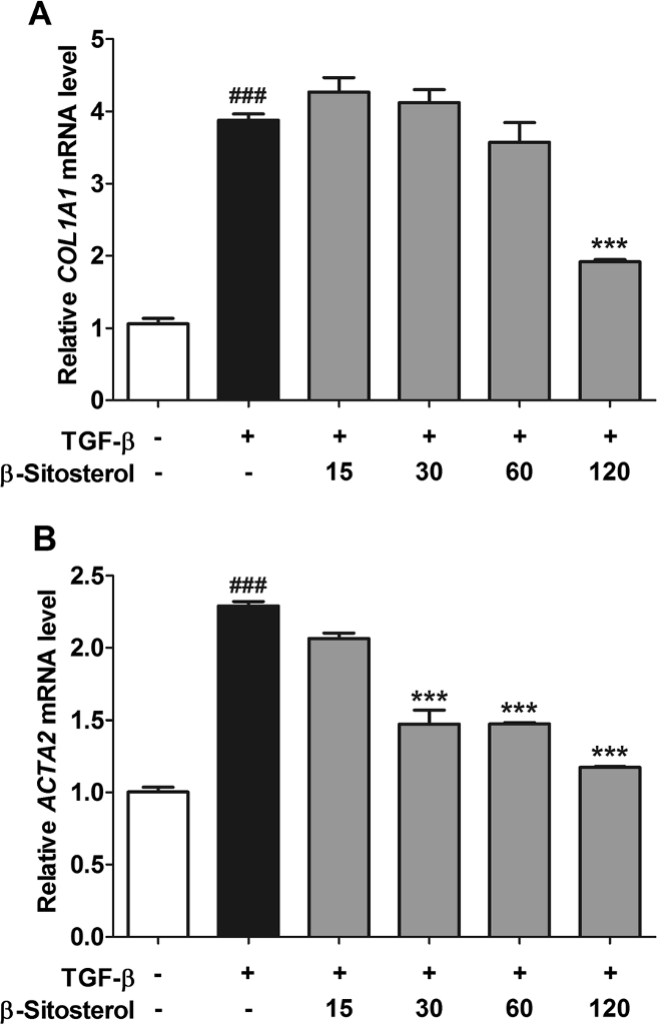
**Effects of** ***β*** **-sitosterol on the collagen-1 and** ***α-SMA*** **mRNA expressions in activated HSCs.** Relatively expressed *COL1A1* **(A)** and *ACTA2* **(B)** levels were measured by real-time quantitative PCR. Experiments were carried out at least twice performed in triplicate. Statistical significance determined by one-way ANOVA; values are means ± SEM; ***, *p* < 0.001 vs TGF-*β-*treated group. ###, *p* < 0.001 vs control group.

**Figure 4:**
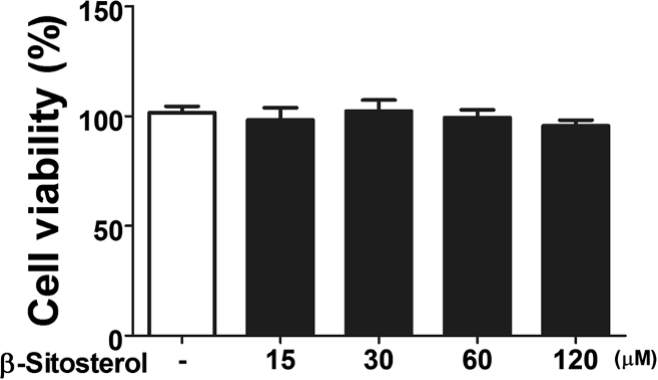
**Cell viability assay of** ***β*** **-sitosterol.** Statistical significance determined by one-way ANOVA; values are means ± SEM.

Total RNAs from the activated HSCs treated by 4-concentrations *β*-sitosterol were isolated and collagen-1 and *α*-SMA mRNA expression levels were measured by real-time PCR (Figure 3). Up regulation of collagen-1 mRNA level triggered by TGF-*β* treatment was prevented by 120 μM of *β*-sitosterol treatment (Figure 3A). And *α*-SMA mRNA level was also prevented by 30-, 60-, and 120 μM of concentration, respectively (Figure 3B). Each concentration of *β*-sitosterol did not affect to the activated LX-2 cell’s viability (Figure 4).

**Figure 5:**
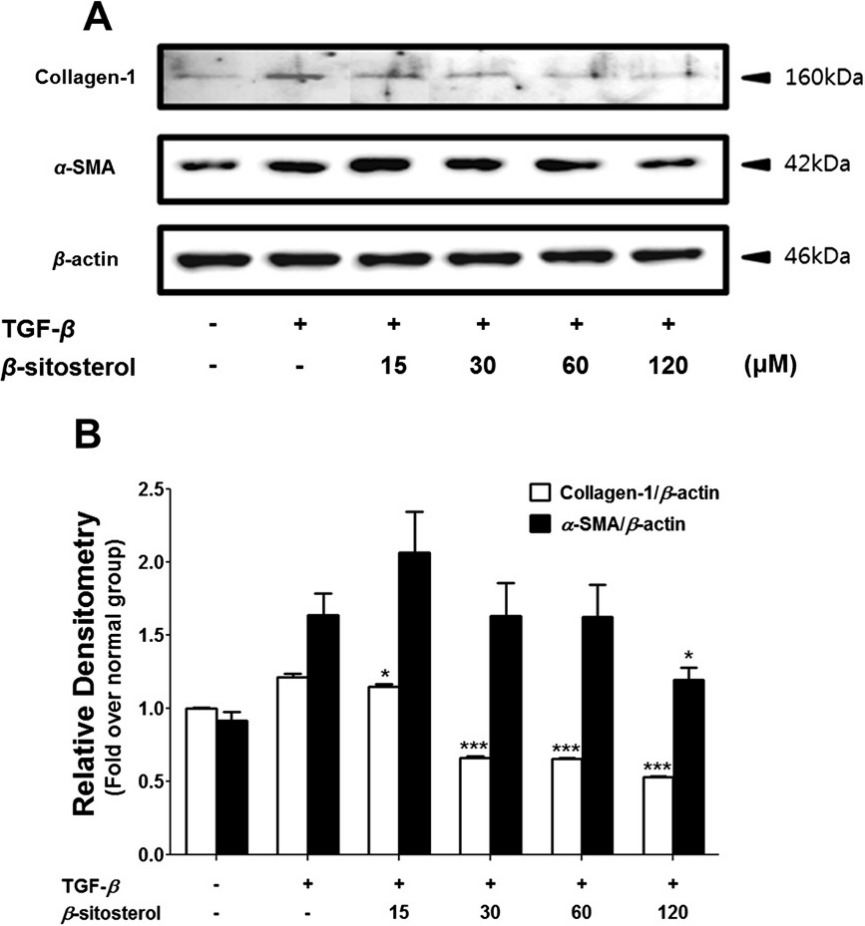
**Effects of** ***β*** **-sitosterol on collagen-1 and** ***α*** **-SMA protein expression in activated HSCs. (A)** The western blot results representative three separate experiments. **(B)** Each protein expressions which normalized by β-actin expression, was measured by densitometry analysis. Statistical significance determined by one-way ANOVA; values are means ± SEM; *, *p* < 0.05; ***, *p* < 0.001 vs TGF-*β-*treated group.

We performed western blot to examine whether the *β*-sitosterol also regulates the collagen-1 and *α*-SMA expression in protein level (Figure 5). TGF-*β* treatment successfully triggered increased expressions of collagen-1 and *α*-SMA levels (Figure 5A). To demonstrate the protein expression levels more concretely, each blotted area was measured and the relative densitometry was translated by bar graphs (Figure 5B). Contrary to the result of collagen-1 mRNA expression level, which only decreased by a highest dose (120 μM), a lowerst dose (15 μM) of *β*-sitosterol was also able to prevent increase of collagen-1 protein expression (Figure 5B). And on the contrary to the result of *α*-SMA mRNA expression level, which decreased by 30 μM of *β*-sitosterol, only 120 μM of the drug affect to the protein expression level (Figure 5B).

### Anti-fibrotie effect of *β*-sitosterol on DMN-induced mouse hepatic fibrosis

**Figure 6:**
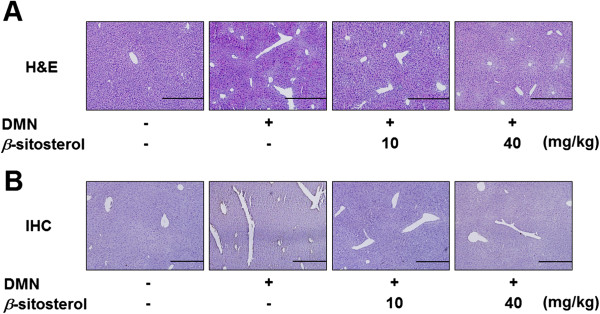
**Effects of** ***β*** **-sitosterol on DMN-induced mouse liver fibrosis. (A)** H&E staining demonstrates the amount of damaged liver tissue. **(B)** The amount of collagen accumulation was determined by IHC.

Hepatic fibrosis mice model was induced by DMN treatment as described at Methods section. After two weeks of drug administration, mouse livers were isolated to determine the anti-fibrotic effect of the *β*-sitosterol. Histological study was performed to 2 different ways those are H&E staining and IHC (Figure 6). The H&E staining was performed to determine whether amount of the DMN-induced liver damage was cured by *β*-sitosterol treatment. The damaged liver tissue area induced by DMN treatment was shown in white gaps (Figure 6A). Two weeks of oral administration of *β*-sitosterol of both concentrations, 10- and 40 mg/kg, reduced the gross area of the damaged tissues (Figure 6A).

The immunostaining experiments using collagen-1 antibody was performed to determine the amount of collagen accumulation on the periphery of the damaged liver tissues. DMN treatment caused the collagen-1 accumulation on the periphery of damaged tissues (Figure 6B). And the IHC results showed DMN-induced accumulation of collagen-1, which showed as brown color, was decreased by oral administration of 10- and 40 mg/kg of *β*-sitosterol (Figure 6B).

### *β*-sitosterol regulates collagen-1 and α-SMA expression levels in DMN-induced mouse hepatic fibrosis

**Figure 7:**
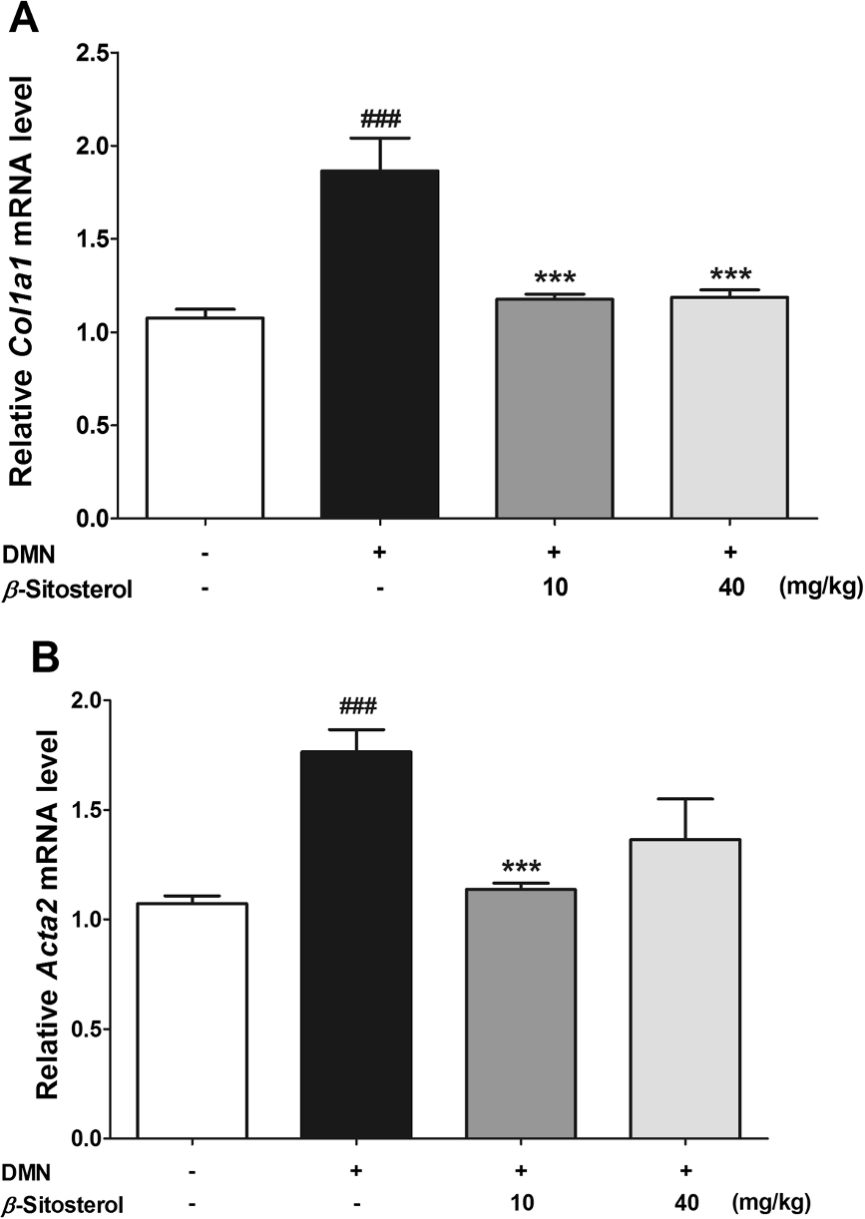
**Effects of** ***β*** **-sitosterol on collagen-1 and** ***α*** **-SMA mRNA expression in DMN-induced mouse liver fibrosis.** Relatively expressed *Col1a1* **(A)** and *Acta2* **(B)** levels were measured by real-time quantitative PCR. Experiments were carried out in triplicate. Statistical significance determined by one-way ANOVA; values are means ± SEM; ***, *p* < 0.001 vs TGF-*β-*treated group. ###, *p* < 0.001 vs control group.

To investigate whether the *β*-sitosterol regulates the collagen-1 and *α*-SMA expression levels *in vivo* as well as in cellular model, we isolated livers of the mouse hepatic fibrosis models treated by saline or each concentration of *β*-sitosterol. We isolated total RNAs to investigate whether *β*-sitosterol regulates the mRNA levels increased by DMN treatment (Figure 7). DMN treatment increased collagen-1 and *α*-SMA mRNA expression levels of mouse liver tissues (Figure 7A and B). Two-weeks of oral administration of the 10 mg/kg of *β*-sitosterol reduced both of the collagen-1 and *α*-SMA mRNA expression level, significantly (Figure 7A and B). However, 40 mg/kg of *β*-sitosterol administration did not affect the *α*-SMA mRNA expression level (Figure 7B).

**Figure 8:**
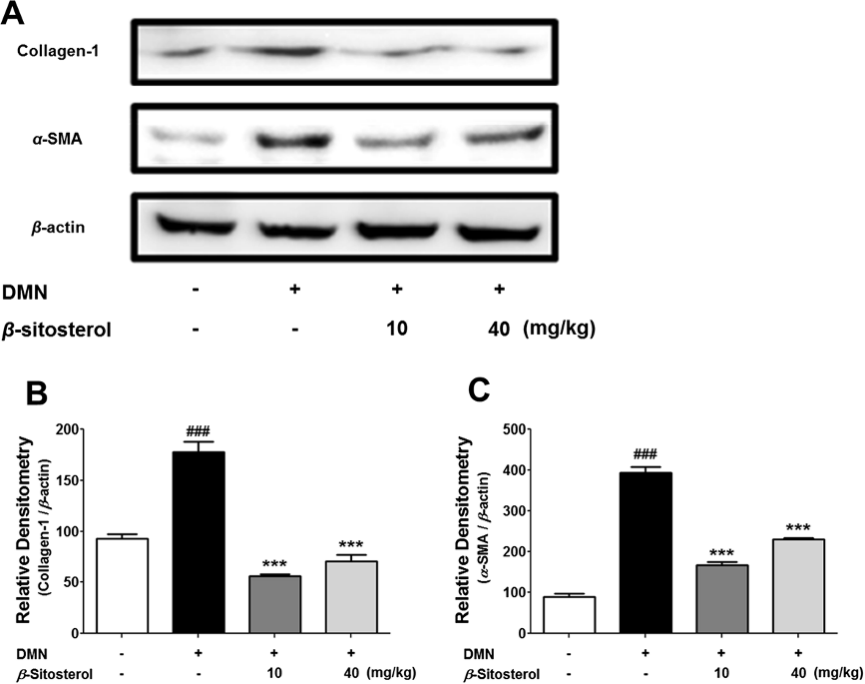
**Effects of** ***β*** **-sitosterol on collagen-1 and** ***α*** **-SMA protein expression in DMN-induced mouse liver fibrosis. (A)** The western blot results representative three separate experiments. **(B-C)** Statistical significance determined by one-way ANOVA; values are means ± SEM; ***, *p* < 0.001 vs TGF-*β-*treated group. ###, *p* < 0.001 vs control group.

We then isolated total proteins from the hepatic fibrosis mice models to investigate the effect of *β*-sitosterol administration on the protein levels (Figure 8). Each blotted area was measured and the relative densitometry was translated by bar graphs (Figure 8B and C). As similar to the real-time PCR results, 10- and 40 mg/kg of *β*-sitosterol reduced both of the collagen-1 and *α*-SMA protein expression levels (Figure 8B and C). But, decrease effect of 40 mg/kg of *β*-sitosterol administration on the protein expression levels was smaller than 10 mg/kg.

## Discussion

Hepatic fibrosis is characterized by scarring due to chronic inflammation from liver diseases. During the process, various cell types are activated and turned into the myofibroblastic cells and then join in synthesis and reorganization of connective tissue [16–18]. A major source of ECM formation in HSC processing is myofibroblastic transition [16, 17]. Myofibroblastic (MFB) differentiation and matrix accumulation of HSC are usually induced by profibrogenic mediators like TGF-*β* and the *β*-isoform of platelet-derived growth factor (PDGF) [16–20].

TGF-*β* secreted by MFB in a latent form leads parenchymal cell (PC) apoptosis [21], stimulates ECM synthesis, provokes the transformation of HSC and elevates ECM production of MFB [19]. Activated HSCs promote not only the synthesis and deposition of the ECM component but also the induction of *α*-SMA. And these signaling cascades accelerate the growth of activated HSCs and contribute to the development of hepatic fibrosis [20]. Therefore, HSC play a key role during fibrosis in response to TGF-*β* through increased synthesis of ECM proteins, especially collagen-I and-II [22].

Several strategies are on trial to break up or reverse hepatic fibrosis. First of all, removing the relevant cause of chronic liver disease is the most effective way to prevent fibrosis. Examples include elimination of excess iron or copper in genetic hemochromatosis or Wilson's disease, abstinence from alcohol, anthelminthic therapy in schistosomiasis, clearance of HBV or HCV in chronic viral hepatitis, and biliary decompression in bile duct obstruction [20, 23–26]. Anti-inflammatory medications may be beneficial in treating fibrosis, because inflammatory mediators may stimulate HSC activation in chronic liver diseases such as viral or autoimmune hepatitis and drug-induced liver injury.

Recently, suppression or reversal of HSC activation has received attention as a therapeutic strategy because of the central role that stellate cells have in fibrogenesis. Gamma interferon, silymarin, fesveratrol, or TGF-*β* antagonists have been noted as examples of suppression HSC activation. And there are several reports of herbal decoctions with anti-fibrotic effects.

AC extract has been studied on the anti-fibrotic and the hepatoprotective effects, and reported to may help liver cells to endure oxidative stress [27–29]. We demonstrated that the active ingredient of AC extract is the *β*-sitosterol (Figure 1), a cholesterol-like phytosterol that widely distributed in the plant kingdom. *β*-sitosterol have been reported to inhibits cholesterol absorption in the intestine and thus reduces blood levels of cholesterol [11].

Here, a novel usage of the *β*-sitosterol was investigated. We induced activated HSC model with LX-2 cells treated by TGF-*β* and confirmed the suitability of the activated HSC model by measuring the mRNA expression levels of HSC activation marker genes, those are MMP-2, Collagen-1, *a*-SMA, and GFAP. These 4 mRNA expressions were up regulated by the TGF-*β* treatment while MMP-1, a collagenase mRNA, was down regulated (Figure 2).

*β*-sitosterol reduced both of collagen-1 and *a*-SMA mRNA expression levels in activated HSC model (Figure 3). Western blot results also show the reducing effect of *β*-sitosterol on the both of collagen-1 and *a*-SMA protein expression levels (Figure 5). On the contrary to the results of the mRNA expression level, the collagen-1 protein expression level was reduced by 15-, 30-, 60-, and 120 μM of *β*-sitosterol treatment (Figure 5A). *α*-SMA protein expression level was also down regulated but only at 120 μM of *β*-sitosterol concentraion (Figure 5A). This un-correlation between the collagen-1 and the *α*-SMA mRNA and protein expression levels may demonstrate the existing of the other modulator for the transcription and translation of the genes. After all the results from cellular model, 120 μM of *β*-sitosterol seems effective concentration to the de-activation of HSCs.

Histological study demonstrated the oral administration of *β*-sitosterol is able to treat the DMN-induced liver damages and to reduce the collagen accumulation around the damaged tissues (Figure 6). This anti-fibrotic effect of the *β*-sitosterol on the DMN-induced mouse hepatic fibrosis is due to the decreasing effect of the drug against the collagen-1 and *a*-SMA expression levels (Figures 7 and 8). Although, 40 mg/kg of *β*-sitosterol did not affect to the *a*-SMA mRNA expression level, it significantly decreased the protein expression. After all the results from *in vivo* model, 10 mg/kg of *β*-sitosterol seems effective to treat the DMN-induced mouse hepatic fibrosis.

## Conclusion

In conclusion, this study demonstrates the effect of *β*-sitosterol, a phytosterol derived from AC water extract, on the collagen and *α*-SMA expression levels in activated HSC model and DMN-induced mouse hepatic fibrosis model. To consider that hepatic fibrosis is closely related to increase of the collagen and *α*-SMA expression levels, regulatory effect of *β*-sitosterol on both of mRNA and protein expressions of the genes demonstrates the drug may be a potential therapeutic agent for the hepatic fibrosis. Although, further pharmacodynamical and toxicological studies are required, our study, using the activated HSCs and *in vivo* model, potentiate the *β*-sitosterol as an anti-hepatofibrosis drug.

## Abbreviations

*α*-SMA: *α-*smooth muscle actin
AC: *Artemisia capillaris*
DMN: Dimethylnitrosamine
ECM: Extracellular matrix
GFAP: Glial fibrillary acidic protein
GAPDH: Glyceraldehyde 3-phosphate dehydrogenase
HSC: Hepatic stellate cell
MMP: Matrix metalloproteinase
MFB: Myofibroblastic
PC: Parenchymal cell
PDGF: Platelet-derived growth factor
TGF-*β*: Transforming growth factor-*β*.
